# Insulin-like growth factor–binding protein 7 is a soluble cell adhesion molecule

**DOI:** 10.1126/sciadv.aeg1148

**Published:** 2026-08-26

**Authors:** Kai Cai, Xuewu Zhang, Xiao-chen Bai

**Affiliations:** ^1^ Department of Biophysics, University of Texas Southwestern Medical Center, Dallas, TX, USA.; ^2^ Department of Pharmacology, University of Texas Southwestern Medical Center, Dallas, TX, USA.; ^3^ Department of Cell Biology, University of Texas Southwestern Medical Center, Dallas, TX, USA.

## Abstract

Insulin-like growth factor–binding protein 7 (IGFBP7) is a secreted protein with diverse roles in angiogenesis, cell differentiation, tissue remodeling, and regulating cell signaling and is linked to numerous human diseases. The molecular basis of the multifunctionality of IGFBP7 remains unclear. Using cryo–electron microscopy, we show that IGFBP7 assembles into a barrel-shaped dodecamer in the presence of heparin. The carboxyl-terminal IgC2 domain forms the central core of the barrel, which is capped by the amino-terminal heparin-binding IB domain at both ends. This homo-oligomer can simultaneously engage heparan sulfate proteoglycans on adjacent cells, functioning as a soluble “cell glue” to drive cell-cell adhesion. Furthermore, IGFBP7 enhances and prolongs signaling of receptor tyrosine kinases, including insulin receptor and c-MET, through its adhesion activity. These findings reveal a structural mechanism for IGFBP7’s pleiotropy and establish it as a universal adhesion factor.

## INTRODUCTION

Insulin-like growth factor–binding protein 7 (IGFBP7), also known as IGFBP-related protein 1 (IGFBP-rP1), MAC25, angiomodulin (AGM), tumor-derived adhesion factor (TAF), and prostacyclin-stimulating factor (PSF), is a secreted 30-kDa protein ubiquitously expressed across many tissues. It regulates a wide range of cellular processes, including angiogenesis, cell differentiation, and tissue remodeling (*1*, *2*). In particular, IGFBP7 is highly expressed in vascular endothelial cells, where it acts as a proangiogenic factor by promoting endothelial adhesion, migration, luminogenesis, capillary morphogenesis, and vessel maturation (*3*–*8*). It also contributes to multiple cell differentiation programs, including stem cell differentiation and epithelial-mesenchymal transition (EMT) (*9*–*13*). Moreover, IGFBP7 is involved in tissue remodeling. It participates in wound healing and tissue repair across diverse organs, including mesenchymal stem cell–mediated lung repair, bone defect healing, keloid formation during skin wound repair, and neurological recovery after traumatic brain injury (*11*, *14*–*16*). Furthermore, IGFBP7 modulates the activities of several key cell surface receptors, including the insulin receptor (IR) (*17*, *18*), insulin-like growth factor 1 receptor (IGF1R) (*19*, *20*), vascular endothelial growth factor (VEGF) receptors (*8*, *21*), transforming growth factor–β (TGF-β) receptors (*22*, *23*), C-type lectin transmembrane receptor CD93 (*24*, *25*), and integrins (*4*).

Given its critical biological functions, dysregulation of IGFBP7 is implicated in numerous diseases, including cancer, heart failure (HF), and acute kidney injury (AKI). IGFBP7 has been linked to a broad spectrum of cancers, such as breast (*26*, *27*), gastric (*28*, *29*), hepatic (*30*, *31*), lung (*32*, *33*), prostate (*34*, *35*), and colorectal (*36*, *37*) cancers, as well as leukemia (*19*, *38*), melanoma (*39*, *40*), and glioma (*41*, *42*), where its role appears to be highly context dependent and influenced by tumor type and microenvironmental cues. In some cancers, IGFBP7 expression is elevated and supports tumor angiogenesis; whereas in others, it shows reduced expression and functions as a tumor suppressor by promoting cellular senescence and apoptosis (*43*, *44*). Beyond cancer, IGFBP7 contributes to pathological cardiac remodeling, cardiomyocyte senescence, and cardiac metabolic dysregulation (*45*–*47*), establishing it as an important regulator of cardiovascular pathology, particularly in HF (*48*–*54*). The American Heart Association has recognized IGFBP7 as one of the most robust biomarkers for HF (*55*). IGFBP7 also plays a key role in the pathophysiology and early detection of AKI (*56*–*59*). Along with tissue inhibitor of matrix metalloproteinases 2 (TIMP-2), IGFBP7 serves as a validated urinary biomarker for AKI risk assessment. The combined biomarker index, [TIMP-2·IGFBP7], commercially known as NephroCheck, is an Food and Drug Administration–approved clinical test used to identify patients at risk of developing moderate to severe AKI (*59*, *60*).

**Fig. 1.**
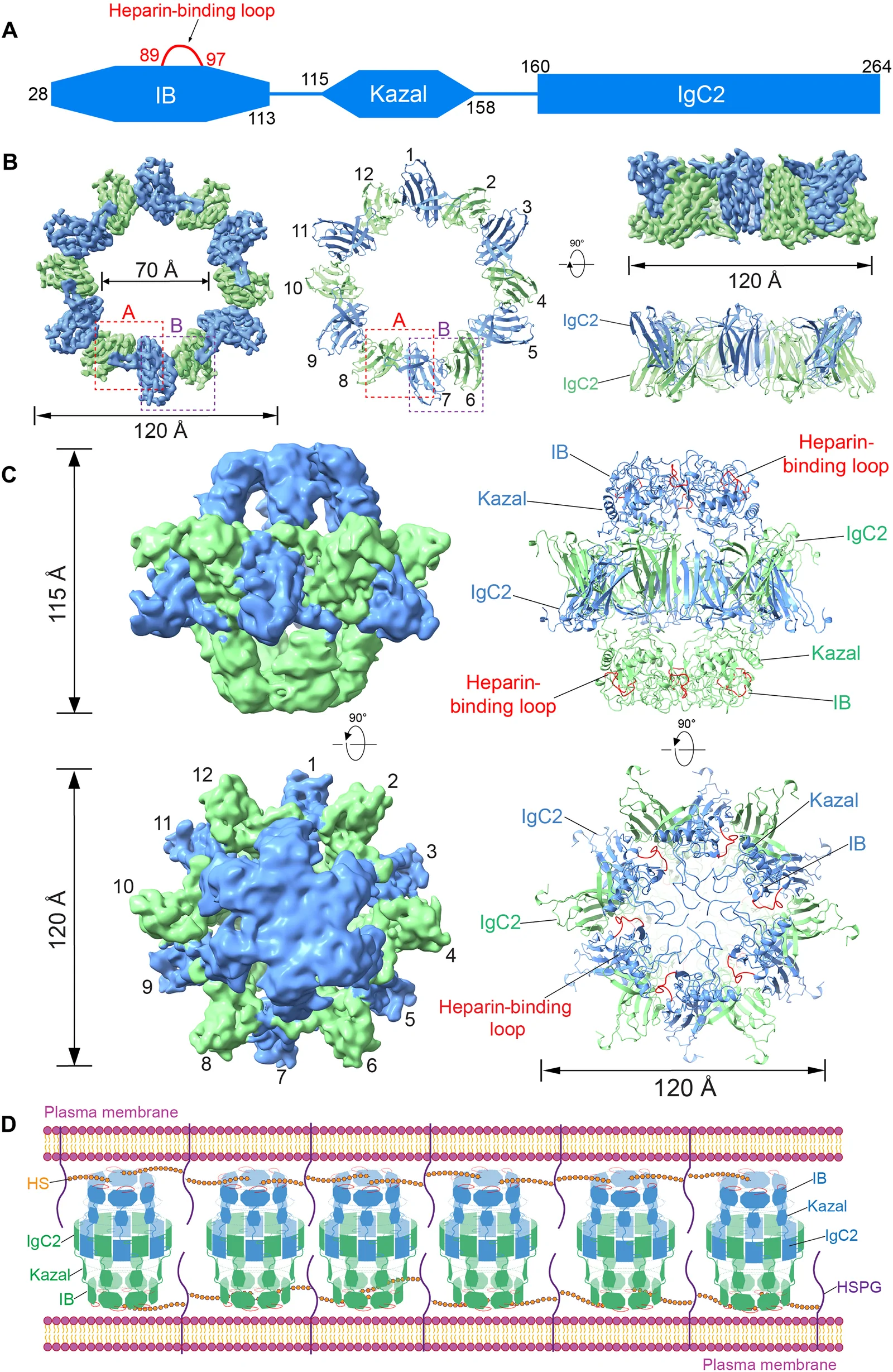
Overall structure of the IGFBP7 dodecamer in complex with heparin. (**A**) Schematic diagram of the domain construct of IGFBP7. IB, IGF binding domain; Kazal, Kazal-type serine protease inhibitor domain; IgC2, C2-type immunoglobulin-like (Ig) domain. (**B**) 3D reconstructions and corresponding ribbon representations of the IGFBP7 dodecamer with a focus on IgC2 domains shown in two orthogonal views. The 3D reconstruction was performed with the D6 symmetry. (**C**) 3D reconstructions and corresponding ribbon representations of the IGFBP7 dodecamer showing the entire protein complex in two orthogonal views. The 3D reconstruction was performed without symmetry enforcement. (**D**) Schematic representation of the IGFBP7 dodecamer in the context of cellular environment. IGFBP7 binds to the heparan sulfate (HS) on the cell membranes and promotes cell adhesion. HSPG, heparan sulfate proteoglycans.

Although IGFBP7 is a member of the IGFBP superfamily, it has several unique features that distinguish it from the classical IGFBPs (IGFBP1–6) (*1*, *61*). Structurally, IGFBP7 comprises three major domains (Fig. 1A): an N-terminal IGF binding domain (IB), a central Kazal-type serine protease inhibitor domain (Kazal), and a C-terminal immunoglobulin-like C2-type domain (IgC2). The IB domain contains a highly positively charged heparin-binding loop, which is essential for IGFBP7’s biological functions (*62*–*64*). Unlike IGFBP1–6, which bind insulin-like growth factors (IGFs) with high affinity, IGFBP7 interacts with both insulin and IGFs only with low affinity (*65*, *66*), indicating that it may function independently of canonical insulin/IGF signaling. Nevertheless, the fundamental molecular mechanisms underlying IGFBP7’s pleiotropic biological activities remain largely undefined.

In this study, we determined the cryo–electron microscopy (cryo-EM) structure of IGFBP7 in the presence of heparin. The structure reveals that heparin binding induces higher-order oligomerization of IGFBP7 into a barrel-shaped dodecameric complex with the D6 symmetry. Notably, cell aggregation assays showed that the IGFBP7 barrel can simultaneously engage heparan sulfate proteoglycans (HSPGs) on adjacent cells, thereby directly promoting cell-cell adhesion independent of classical cell surface receptors. Moreover, cell-based assays demonstrated that IGFBP7 enhances and prolongs the activation of both IR and c-MET receptors through its adhesion-mediated mechanism. Together, our structural and functional findings identify IGFBP7 as a “cell glue” molecule and provide insight into how it achieves functional pleiotropy through its adhesion activity.

## RESULTS

### IGFBP7 forms a barrel-shaped dodecamer in the presence of heparin

To understand how IGFBP7 functions, we expressed human IGFBP7 in human embryonic kidney (HEK) 293 cells and purified it as a secreted protein (fig. S1). Previous studies have shown that IGFBP7 binds heparin through a highly positively charged loop within its IB domain (*64*). We quantitatively characterized the interactions between IGFBP7 and heparins using biolayer interferometry (BLI), in which biotinylated heparins were immobilized on streptavidin (SA) biosensors. Three heparin polymers of different chain lengths were tested: a long-chain heparin with an average molecular mass of 15 kDa, referred to as LCHS; a heparin dodecasaccharide with a defined molecular mass, referred to as 12HO; and tinzaparin. The measured dissociation constants (*K*_d_) for IGFBP7 binding to LCHS, 12HO, and tinzaparin were 46.7, 142.9, and 163.9 nM, respectively (fig. S2).

Notably, both size exclusion chromatography (SEC) and SEC coupled with multiangle light scattering (SEC-MALS) revealed that IGFBP7 predominantly exists as a monomer in the absence of heparin, whereas heparin binding promotes the formation of large, higher-order IGFBP7 oligomers (figs. S3 and S4). Although all three types of heparin were able to induce higher-order IGFBP7 oligomerization, LCHS showed a substantially greater capacity to promote oligomerization than either 12HO or tinzaparin (figs. S3 and S4). Therefore, LCHS was selected for all subsequent experiments.

We then sought to determine the cryo-EM structure of IGFBP7 in the presence of heparin (fig. S5). The two-dimensional (2D) class averages revealed a distinct ring-like particle shape (fig. S5). The cryo-EM reconstruction reached an overall resolution of 3.4 Å, revealing a barrel-shaped homo-dodecamer with D6 symmetry (Fig. 1B). The inner and outer diameters of the ring are ∼70 and 120 Å, respectively. The 12 IgC2 domains of IGFBP7 were well resolved, allowing for precise model building based on clear side-chain densities. The structure shows that two IgC2 domains form an antiparallel dimer, and six such dimers assemble in a circular manner to form the complete dodecameric ring (Fig. 1B). The detailed interaction patterns would be described in the following sections.

After further 3D classification with symmetry expansion, the N-terminal IB domain and the central Kazal domain from all 12 IGFBP7 subunits were resolved at an overall resolution of 4.5 Å (Fig. 1C). Despite the moderate resolution, the AlphaFold-predicted structures of the IB and Kazal domains could be rigidly fitted into the cryo-EM density unequivocally, guided by well-defined secondary structural features. This enabled the construction of a complete model for the IGFBP7 homo-dodecamer (Fig. 1C). The resulting structure resembles a barrel ∼115 Å in height, in which the IB and Kazal domains of the 12 subunits alternately point in opposite directions. Consequently, six IB/Kazal domains are positioned in each half of the barrel. Intriguingly, the six heparin-binding loops within the IB domains cluster at the top and bottom surfaces of the barrel. Although heparin itself was not resolved in the cryo-EM map, the close proximity of these positively charged loops suggests that a single heparin polymer could simultaneously engage multiple heparin-binding loops, thereby stabilizing the barrel assembly (Fig. 1, C and D). This structural arrangement therefore provides a mechanistic explanation for how heparin binding induces higher-order oligomerization of IGFBP7 (Fig. 1D). Furthermore, the clustering of highly positively charged heparin-binding loops on both ends of the IGFBP7 barrel likely facilitates its interaction with cell surface HSPGs (Fig. 1D). In contrast to the tightly packed IgC2 domains, the neighboring Kazal domains do not make contacts with one another, whereas the IB domains make packing interactions at the two ends of the barrel, although the details of these interactions are not well resolved in the cryo-EM map.

### Detailed assembly mechanism of the IGFBP7 barrel

**Fig. 2.**
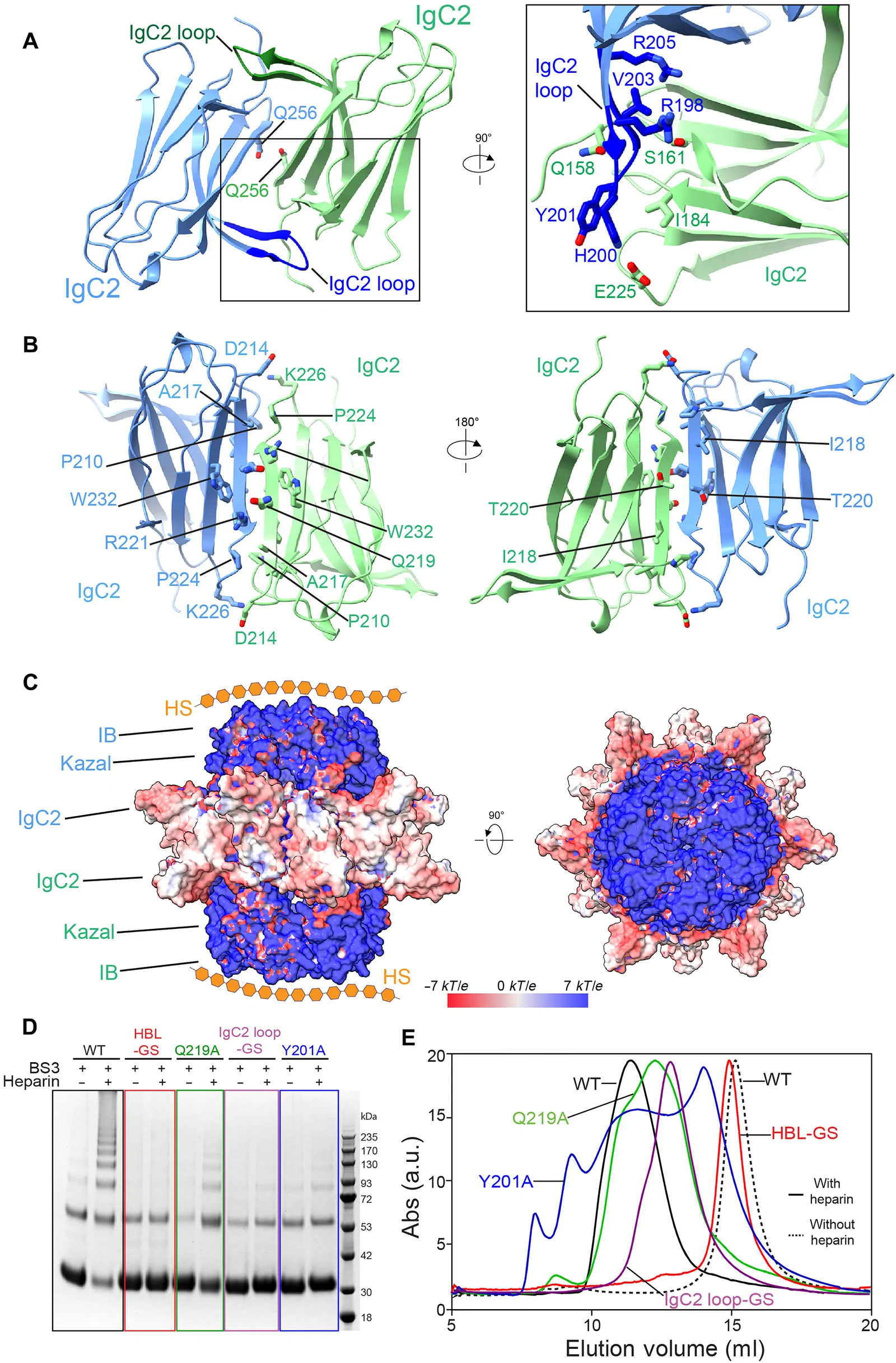
Structural details of the IGFBP7 dodecamer. (**A**) Type A interface of two IgC2 domains from two adjacent IGFBP7 in the dodecamer. The key feature of this interface is the engagement of the extended interstrand IgC2 loop (highlighted with darker green or blue colors) with the neighboring IgC2 domain. Right: Close-up view of the boxed area in the left showing the detailed interaction between IgC2 loop and the neighboring IgC2 domain. The key residues involved in the interaction are highlighted. (**B**) Type B interface of two IgC2 domains from two adjacent IGFBP7 in the dodecamer shown in two views. The key feature of this interface is the formation of an extended and twisted β sheet. Key residues involved in the interaction are highlighted. (**C**) Surface representation of the IGFBP7 dodecamer showing the charge distribution in two orthogonal views. The surface is colored on the basis of electrostatic potential calculated using the APBS server. HS binds to the highly positively charged surface formed by heparin-binding loops in the IB domains. (**D**) BS3 cross-linking assay for WT and mutant IGFBP7 in the absence or presence of heparin. (**E**) SEC profiles of IGFBP7 WT and mutants without or with heparin. a.u., arbitrary units.

The central part of the barrel of the IGFBP7 dodecamer consists of a ring made up of 12 IgC2 domains, whereas the IB and Kazal domains extend from each side of this IgC2 ring (Fig. 1C). We observed two distinct types of direct IgC2-IgC2 interactions that are essential for maintaining the IgC2 ring, designated as type A and type B (Fig. 1B). At the type A interface, a long interstrand loop from one IgC2 domain engages the side surface of the adjacent IgC2 domain, forming a key contact interface (Fig. 2A). Residues Arg^198^, His^200^, Tyr^201^, Val^203^, and Arg^205^ from one IgC2 interact with Gln^158^, Ser^161^, Ile^184^, and Ile^186^ from the other (Fig. 2A). Notably, this interstrand loop within the IgC2 domain adopts a distinct conformation compared with the structure predicted by AlphaFold (*67*) (fig. S3D), suggesting an induced-fit mechanism in which the loop undergoes a conformational change upon dimerization. In addition, two Gln^256^ residues from opposing protomers form homotypic contacts that further stabilize this interface (Fig. 2A).

The type B interface is primarily formed through the assembly of an extended antiparallel β sheet between the IgC2 domains of two adjacent IGFBP7 protomers, complemented by additional contacts involving interstrand loops (Fig. 2B). This interaction creates an eight-stranded, twisted β sheet that spans the center of the dimeric assembly. The interface is mainly stabilized by hydrogen bonds involving both main-chain and side-chain atoms. For example, Gln^219^ and Arg^221^ from one IgC2 domain form hydrogen bonds with the same residues from the opposing IgC2 domain (Fig. 2B). In addition, Ala^217^ forms a hydrophobic contact with Pro^224^ between neighboring subunits, further stabilizing this interface (Fig. 2B).

The six N-terminal IB/Kazal domains, together with the heparin-binding loops, are positioned at the top and bottom of the IGFBP7 dodecameric barrel (Fig. 2C). Similar to other IGFBPs, the IB domain of IGFBP7 consists of extensive loop regions stabilized by six disulfide bridges (fig. S6). The six IB domains are arranged at the extreme top and bottom of the IGFBP7 barrel, forming two flat and highly positively charged rings (Fig. 2C). Loops from neighboring IB domains appear to interact with one another, although the details of these interactions are not clearly resolved due to poor cryo-EM density in this area (fig. S5). Nonetheless, these interdomain associations are likely reinforced by the bound heparin chains, which may serve as molecular tethers by simultaneously engaging multiple heparin-binding loops, leading to stable IB domain rings at both ends of the IGFBP7 dodecameric barrel (Fig. 2C).

### Validation of the structural model using cross-linking

To validate the observed oligomerization model of IGFBP7, we introduced mutations to key residues at the IgC2-IgC2 interfaces. In particular, Tyr^201^–>Ala (Y201A) for the type A interface and Gln^219^–>Ala (Q219A) for the type B interface (Fig. 2, A and B). In addition, we replaced the interstrand loop in the IgC2 domain (referred to as the IgC2 loop hereafter) and the heparin-binding loop in the IB domain (referred to as HBL hereafter) with flexible glycine-serine (G-S) linkers, namely, IgC2 loop-GS and HBL-GS, respectively. Similar to wild-type (WT) IGFBP7, all four mutant proteins could be expressed in HEK293 cells and purified as secreted proteins. In the absence of heparin, all four mutants predominantly existed as monomers (figs. S1 and S7). We assessed the oligomerization propensities of these IGFBP7 mutants using bis(sulfosuccinimidyl) suberate (BS3)–mediated cross-linking, SEC, and SEC-MALS. The cross-linking assays showed that WT IGFBP7 could be cross-linked into dimers in the absence of heparin and assembled into large higher-order oligomers upon heparin addition (Fig. 2D). In contrast, the mutant proteins displayed either abolished or markedly reduced oligomerization under the same conditions (Fig. 2D). Consistently, SEC and SEC-MALS analyses showed that all IGFBP7 mutants exhibited abolished or reduced capacity to form higher-order oligomers, supporting our structural model (Fig. 2E and fig. S7).

Moreover, we used BLI to quantitatively analyze the interactions between heparin and the IGFBP7 mutants. Whereas the HBL-GS mutant showed no detectable heparin binding, the other three mutants all exhibited reduced binding affinities compared with WT IGFBP7 (fig. S8). These results suggest a reciprocal mechanism in which heparin induces higher-order oligomerization of IGFBP7, and higher-order oligomerization, in turn, strengthens heparin binding.

### IGFBP7 induces cell-cell adhesions and concentrates at cell-cell junction sites

In our structural model of the barrel-shaped IGFBP7 homo-dodecamer, both the top and bottom surfaces of the barrel contain clusters of six heparin-binding loops capable of engaging heparin molecules efficiently (Figs. 1C and 2C). This arrangement suggests that the IGFBP7 barrel could potentially mediate cell-cell adhesion by simultaneously binding to HSPGs on the surfaces of two adjacent cells (Figs. 1D and 2C). In this configuration, multiple IGFBP7 barrels could be sandwiched between two neighboring cells (Fig. 1D). Consistent with this model, early studies reported that IGFBP7 has adhesion-promoting activity (*3*, *68*).

**Fig. 3.**
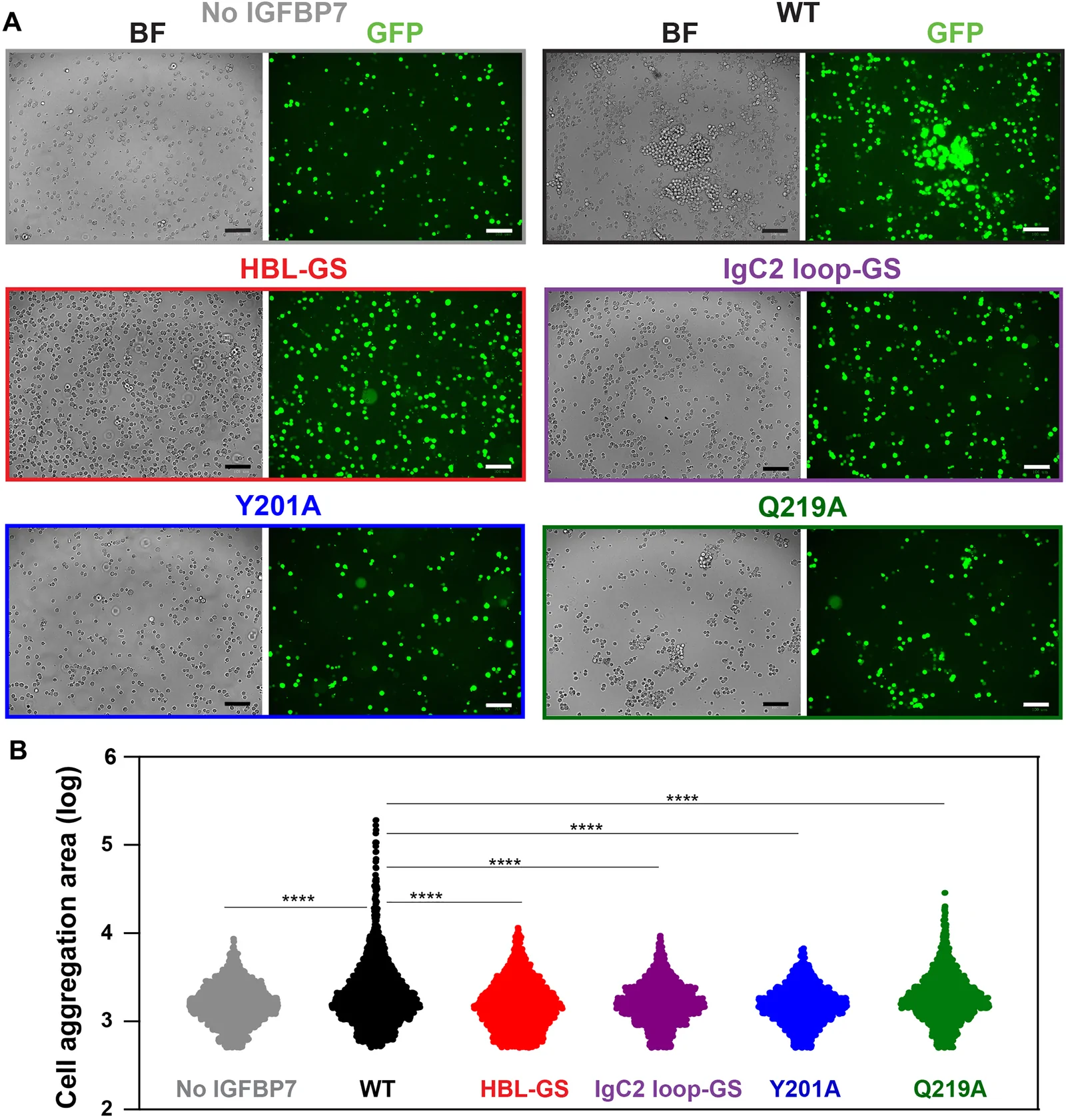
Cell aggregation experiments on HEK293T cells show that IGFBP7 promotes cell aggregation. (**A**) Representative microscopic images of HEK293T cells without or with the addition of IGFBP7 WT or mutants. HEK293T cells were transfected with pcDNA3.1+GFP for better visualization. Robust cell clustering was observed in the presence of WT IGFBP7 (top right) but not in the absence of IGFBP7 (top left) or with indicated IGFBP7 mutants (middle and bottom). BF, bright field; GFP, GFP fluorescence. Scale bars, 100 μm. (**B**) Quantification of cell aggregation by calculating the aggregated areas of 30 images. ****P < 0.0001.

To test this hypothesis, we assessed the adhesive function of IGFBP7 using cell aggregation and cell-cell junction enrichment assays. In the cell aggregation assay, HEK293T cells expressing green fluorescent protein (GFP) were incubated with WT or mutant IGFBP7 proteins at a concentration of 200 nM, and cell aggregation was subsequently imaged and quantified. WT IGFBP7 efficiently induced cell aggregation, whereas the mutants that disrupt either the type A IgC2-IgC2 dimer interface (IgC2 loop-GS or Y201A) or heparin binding (HBL-GS) failed to do so (Fig. 3, A and B). The Q219A mutant was still able to promote cell aggregation but with reduced efficiency compared to WT (Fig. 3, A and B). These results demonstrate that IGFBP7 functions as a soluble adhesion molecule that mediates cell-cell adhesion through interactions with cell surface HSPGs. Furthermore, the heparin-induced oligomerization of IGFBP7 is essential for its adhesive activity.

**Fig. 4.**
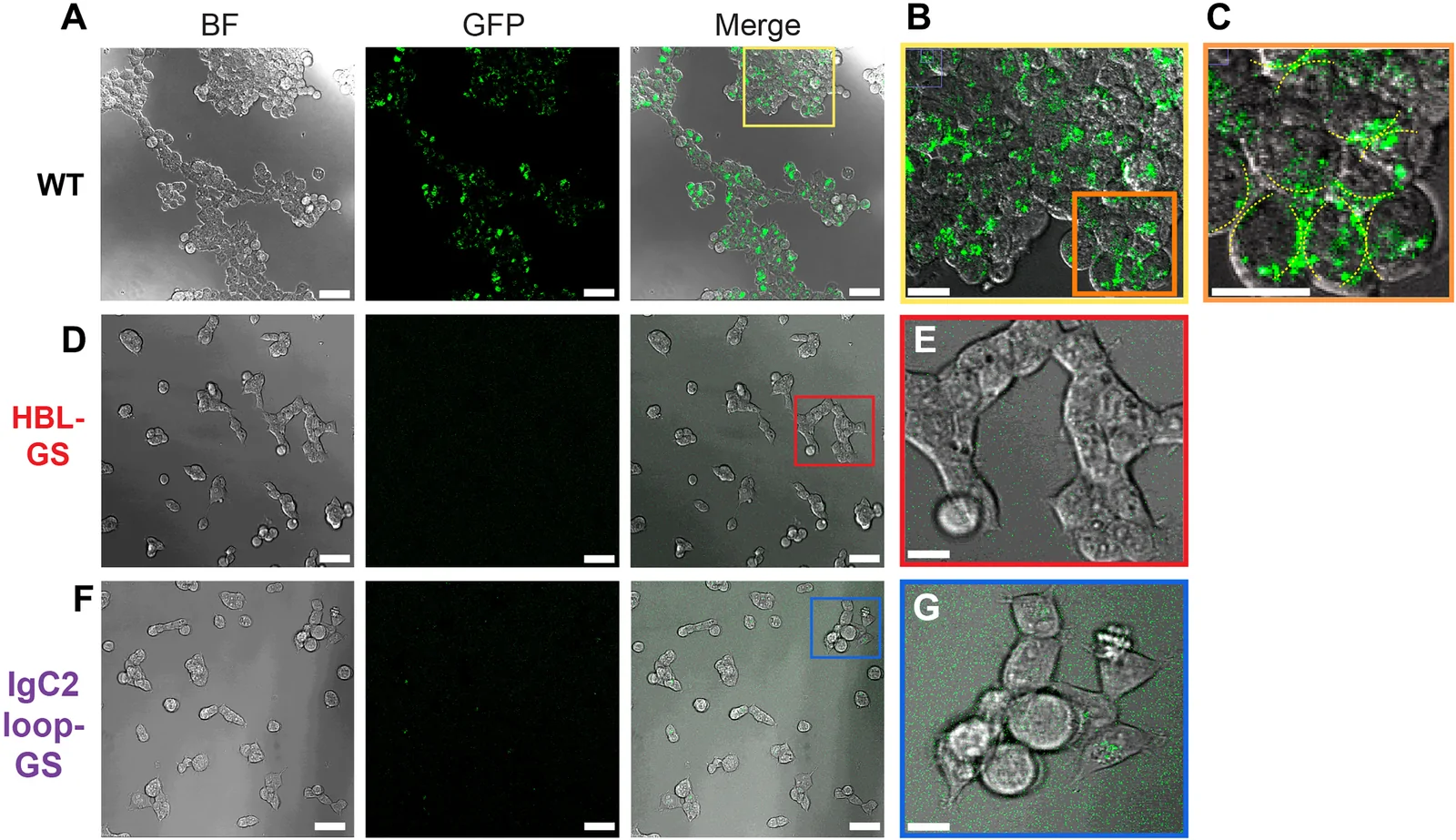
IGFBP7 binds to the cell surface and enriches at the cell-cell interfaces. HEK293T cells were treated with purified WT or mutants of IGFBP7-eGFP for 8 hours. Cells were washed twice with DPBS to remove unbound IGFBP7-eGFP before imaging. (**A**) Representative microscopic images of HEK293T cells after treatment with IGFBP7-eGFP. Strong GFP fluorescence signal was observed with WT IGFBP7, indicating that WT IGFBP7 was capable of binding to the cell surface. BF, bright field; GFP, GFP fluorescence. (**B**) Close-up view of the region indicated by the yellow box in (A). (**C**) Close-up view of the region indicated by the orange box in (B). Yellow dotted lines denote the boundaries between HEK293T cells. (**D**) Representative microscopic images of HEK293T cells after treatment with of the IGFBP7-eGFP heparin-binding loop mutant (HBL-GS). (**E**) Close-up view of the region indicated by the red box in (D). (**F**) Representative microscopic images of HEK293T cells after treatment with the IGFBP7-eGFP IgC2 loop mutant (IgC2 loop-GS). (**G**) Close-up view of the region indicated by the blue box in (F). Scale bars, 50 μm [for (A), (D), and (F)] and 20 μm [for (B), (C), (E), and (G)].

In the cell-cell junction enrichment assay, HEK293T cells were incubated with recombinant GFP-fused WT or mutant IGFBP7 proteins at a concentration of 200 nM. Microscopic images revealed that WT IGFBP7 efficiently bound to the surface of HEK293T cells, localized to cell-cell junctions, and exhibited a clustered distribution pattern (Fig. 4, A to C). In contrast, the IgC2 loop-GS and HBL-GS mutants showed markedly reduced binding to the cell surface. In addition, these mutants failed to form discernible puncta on the surface of HEK293T cells (Fig. 4, D to G). Together, these findings indicate that IGFBP7 accumulates at cell-cell junctions, where it promotes cell adhesion, consistent with our proposed model (Fig. 1D).

### The cell adhesion induced by IGFBP7 promotes the signaling of receptor tyrosine kinase

**Fig. 5.**
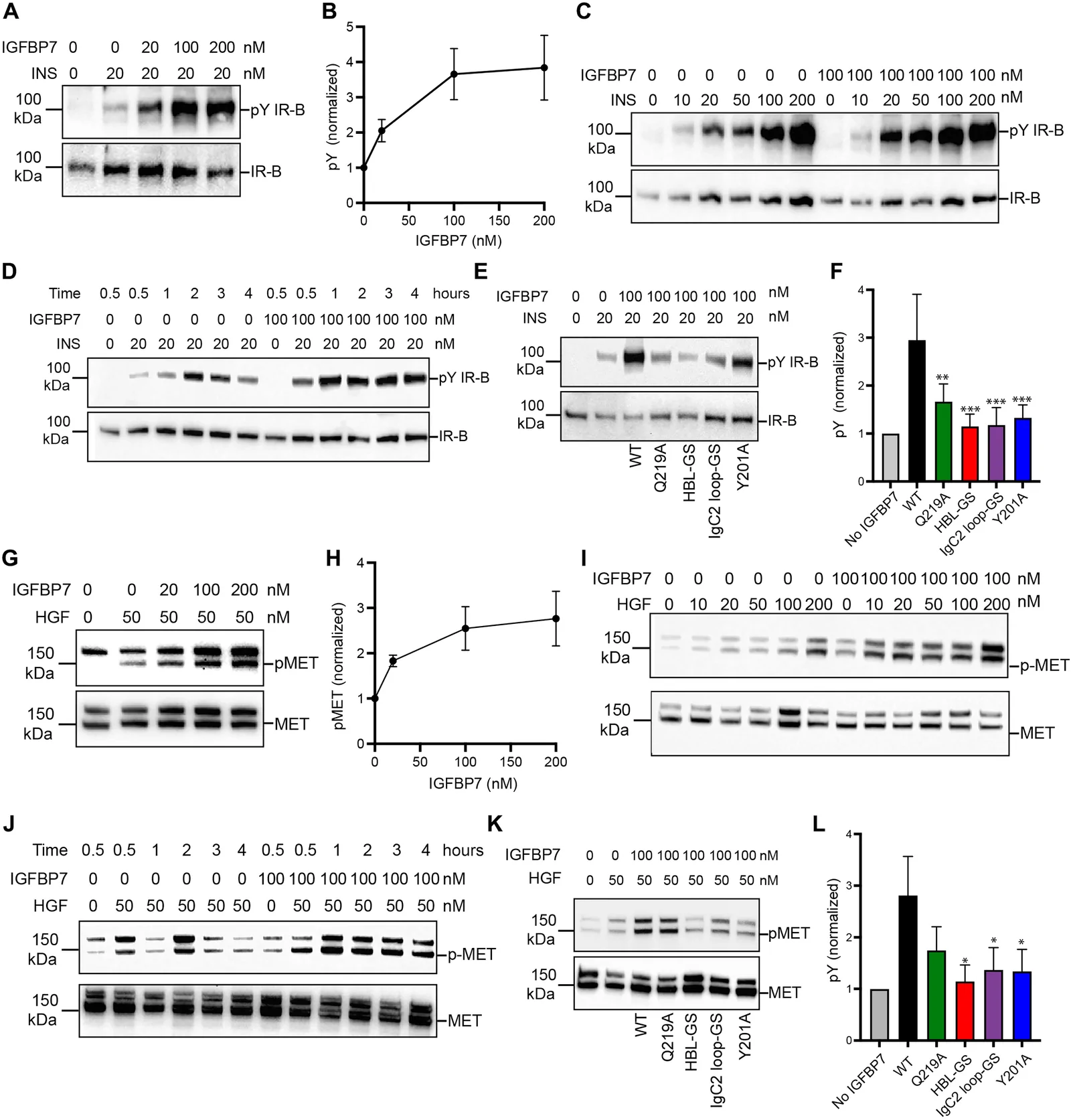
IGFBP7 promotes IR and c-MET receptor signaling. (**A** to **F**) Western blotting images of the autophosphorylation of IR induced by insulin (INS) in HEK293T cells expressing WT IR under indicated conditions. (**A**) Western blot analysis of IR autophosphorylation in the presence of increasing concentrations of WT IGFBP7. IGFBP7 enhances IR autophosphorylation in a dose-dependent manner. (**B**) Quantification of the Western blot data shown in (A). The experiment was repeated four times. (**C**) Western blot analysis of IR autophosphorylation in the presence of increasing concentrations of INS and in the presence or absence of 100 nM WT IGFBP7. (**D**) Time course of INS-stimulated IR autophosphorylation in the absence or presence of 100 nM IGFBP7. IGFBP7 prolongs the duration of IR activation. (**E**) INS-stimulated IGF1R autophosphorylation with or without 100 nM WT IGFBP7 or the indicated IGFBP7 mutants. (**F**) Quantification of the Western blot data shown in (E). The experiment was repeated eight times. Statistical significance was calculated using a two-tailed Student’s *t* test; between WT and mutant; **P < 0.01 and ***P < 0.001. (**G** to **L**) Western blotting images of the autophosphorylation of c-MET induced by HGF under indicated conditions. (G) Western blot analysis of c-MET autophosphorylation in the presence of increasing concentrations of WT IGFBP7. (H) Quantification of the Western blot data shown in (G). The experiment was repeated four times. (I) c-MET autophosphorylation induced by various concentrations of HGF with or without the presence of 100 nM of WT IGFBP7. (J) Time course of HGF-induced c-MET autophosphorylation in the absence or presence of 100 nM IGFBP7. (K) HGF-stimulated c-MET autophosphorylation with or without 100 nM WT IGFBP7 or the indicated IGFBP7 mutants. (L) Quantification of the Western blot data shown in (K). The experiment was repeated three times. Statistical significance was calculated using a two-tailed Student’s *t* test; between WT and mutant; *P < 0.05.

Previous studies have shown that IGFBP7 modulates the activation of a wide range of cell surface receptors, including the IR (*17*, *18*), IGF1R (*19*, *20*), VEGFR (*8*, *21*), TGF-β receptors (*22*, *23*), integrins (*4*), and CD93 (*24*, *25*) and their downstream signaling pathways. We hypothesized that these regulatory activities may be mediated through its cell adhesion function. To test this idea, we performed cell-based assays to examine the effect of IGFBP7 on IR signaling. Our results showed that cotreatment of insulin and IGFBP7 induced higher levels of IR phosphorylation than insulin alone and that IGFBP7 enhanced insulin-stimulated IR activation in a dose-dependent manner (Fig. 5, A to C). Time-course experiments further revealed that insulin-induced IR signaling is normally transient, with the signaling amplitude starting to reduce within 3 hours of stimulation. The presence of IGFBP7 substantially prolonged IR activation, converting the transient response into a more sustained signaling (Fig. 5D). Mutations that disrupt IGFBP7 oligomerization or heparin binding markedly reduced or abolished this effect, confirming that IGFBP7 promotes insulin-induced IR signaling through an adhesion-mediated mechanism (Fig. 5, E and F).

c-MET is the receptor tyrosine kinase that is activated by hepatocyte growth factor (HGF) (*69*, *70*). Similar to its effect on IR, WT IGFBP7 enhanced HGF-induced c-MET activation in a dose-dependent manner and prolonged the duration of signaling (Fig. 5, G to J). In contrast, IGFBP7 mutants lacking the ability to form oligomers or bind heparin exhibited reduced or abolished effects on c-MET activation (Fig. 5, K and L). Together, these findings demonstrate that IGFBP7 broadly regulates the activation of cell surface receptors through its cell adhesion activity.

## DISCUSSION

Here, by using cryo-EM and biochemical analyses, we show that IGFBP7 forms a barrel-shaped homo-dodecamer with D6 symmetry in the presence of heparin. The overall architecture of the IGFBP7 barrel resembles that of the T-complex protein Ring Complex (TRiC) (*71*). Oligomerization is driven by both IgC2 domain–mediated interactions and the binding of heparin to the heparin-binding loops within the N-terminal IB domains. We demonstrate that this structural arrangement enables an IGFBP7 oligomer to bind HSPGs on two opposing cell surfaces simultaneously, thereby promoting cell-cell adhesion. Last, we show that this adhesion activity is essential for previously described biological functions of IGFBP7, including the modulation of receptor tyrosine kinase signaling.

We identified two distinct types of IgC2-IgC2 interfaces, with one involving a long interstrand loop (IgC2 loop) that mediates IGFBP7 dimerization (type A), and the other stabilizing the β sheet packing between adjacent dimers (type B). Six IB domains, along with their heparin-binding loops, are arranged on the opposite ends of the IGFBP7 barrel. The ability of a single heparin polymer to engage multiple heparin-binding loops further stabilizes the barrel-shaped oligomer. Previous studies have shown that the heparin-binding loop is essential for the functions of IGFBP7 regulated by serine proteinase matriptase and RNA editing (*62*–*64*). Sequence alignment of IGFBP7 across species shows that both IgC2 and heparin-binding loops are among the least conserved regions within IGFBP7 proteins (fig. S9). These regions are not fully conserved even among vertebrates, suggesting that IGFBP7 may have distinct biological functions in lower species.

In both SEC and SEC-MALS experiments, the elution peak of IGFBP7 progressively shifted to earlier fractions with increasing amounts of heparin. In addition, the SEC-MALS profiles of IGFBP7 in the presence of different amounts of heparin indicate a high degree of sample heterogeneity, with a broad range of oligomeric species ranging from monomers to very high-order oligomers. These results support the presence of heparin-induced intermediate oligomeric assemblies. We speculate that the intermediate IGFBP7 oligomers may contain 2 to 11 IGFBP7 protomers, assembled through IgC2-IgC2 interactions similar to those observed in the IGFBP7 dodecamer structure. However, these intermediate oligomeric states are likely structurally flexible and heterogeneous, which may explain why they could not be resolved by cryo-EM analyses.

We showed that IGFBP7 modulates both the amplitude and duration of signaling of two RTKs, IR and c-MET. Mutations that disrupt IGFBP7 oligomerization or its interaction with heparin lead to diminished or abolished regulatory effects, indicating that IGFBP7 controls RTK signaling through its cell adhesion activity rather than through direct binding to the receptors or their ligands. Nevertheless, the precise mechanism by which IGFBP7 enhances signaling remains unclear. One plausible explanation is that IGFBP7-induced cell-cell adhesion may impede receptor internalization via endocytosis, thereby sustaining receptor presence on the cell surface and prolonging signaling. Moreover, it is worth noting that, through heparin binding, IGFBP7 could, in principle, oligomerize and cluster on the membrane of an individual cell, thereby coating the cell surface. In this manner, IGFBP7 may alter membrane curvature and fluidity, which could indirectly modulate cell surface receptor signaling in individual cells independent of cell-cell adhesion. These possibilities warrant further investigation in future studies.

In addition to regulating cell surface receptor signaling, IGFBP7 is a versatile protein implicated in numerous biological processes, including angiogenesis, tissue development, cell differentiation, and tissue remodeling (*1*). Because cell-cell adhesion is a fundamental component for all these processes, we propose that IGFBP7 may regulate these biological functions through the same adhesion-based mechanism. The ability of IGFBP7 to induce adhesion across diverse cell types, which is simply through self-oligomerization and heparin binding, likely underlies its broad functional versatility.

To date, most well-characterized cell adhesion events are mediated either by homotypic or heterotypic interactions between single transmembrane adhesion molecules, or by interactions between single transmembrane adhesion receptor and adhesion G protein–coupled receptors (GPCRs) (*72*, *73*). In contrast, IGFBP7 is a soluble molecule capable of directly inducing cell adhesion through self-oligomerization and binding to cell surface HSPGs, without requiring engagement of any dedicated cell surface receptors. Given that HSPGs are universally expressed on the surface by nearly all eukaryotic cells and are abundant within the extracellular matrix, it is intriguing to consider that IGFBP7 may function as a broadly acting “universal cell glue” that is capable of promoting adhesion across diverse cell types. The extent to which IGFBP7 can induce cell adhesion is likely determined by its circulating concentration and the abundance of HSPGs on the cell surface.

IGFBP7 has been implicated in numerous human diseases. In addition to the abovementioned diseases including AKI, HF, and cancer, recent studies have identified IGFBP7 as either a marker or contributing factor in a broad spectrum of other diseases, such as idiopathic pulmonary fibrosis (IPF), acute lung injury (*23*, *74*), and insulin resistance (*17*, *75*, *76*). The involvement of IGFBP7 in such a diverse array of pathological conditions is consistent with its functional pleiotropy and regulatory versatility. On the basis of our structural model, therapeutic strategies aimed at disrupting IGFBP7 oligomerization or blocking its interaction with heparin, such as through antibodies or engineered protein binders, may hold substantial promise. Moreover, given the possibility that IGFBP7 functions as a universal cell-cell “glue,” it could also serve as a valuable tool for probing the functional relevance of cell-cell adhesion in diverse biological contexts.

## MATERIALS AND METHODS

### Constructs design

The human cDNA encoding full-length IGFBP7 was cloned into pEZT-BM expression vector using Gibson assembly reagents (New England Biolabs). The Human Rhinovirus 3C recognition site, affinity purification tag Tsi3 (T6SS secreted immunity protein three from *Pseudomonas aeruginosa*) and His8 tag were fused to the C terminus of proteins as described previously (*77*). All IGFBP7 mutants were generated using site-directed mutagenesis (Q5 Site-Directed Mutagenesis, New England Biolabs). The WT and mutant IGFBP7-eGFP constructs were also cloned into pEZT-BM expression vector using Gibson assembly reagents.

### Protein expression and purification

IGFBP7 (WT and mutants) and IGFBP7-eGFP (WT and mutants) proteins were expressed in HEK293F cells using polyethylenimine (PEI) transient transfection following a published protocol (*78*) with modifications. Briefly, the plasmids were mixed with PEI MAX (Polysciences) in Opti-MEM reduced serum media (Gibco) at a ratio of 1:3 (DNA:PEI, w/w) for 30 min in a Laminar flow hood, and the plasmid/DNA mix was added to HEK293F cells cultured to about 2.5 × 10^6^ cells/ml in Gibco FreeStyle 293 media (Thermo Fisher Scientific). After 16 hours, the infected cells were supplemented with 5 mM sodium butyrate (Sigma-Aldrich) to increase protein expression. Cells were cultured in a shaking incubator for 72 hours at 37°C and 8% CO_2_ before harvesting.

The purification of WT and mutant IGFBP7 performed in the same way. Briefly, culture media containing the secreted IGFBP7 was harvested by centrifugation and filtered using a glass fiber filter. The clarified media were incubated with the INDIGO-Ni resin (Cube Biotech) at 4°C for 1 hour on the bottle roller. The resin was collected using a chromatography column by gravity flow. The resin was then washed with 10 resin volumes of the washing buffer [20 mM Hepes (pH 7.5), 150 mM NaCl, and 20 mM imidazole] and eluted with the elution buffer [20 mM Hepes (pH 7.5), 150 mM NaCl, and 300 mM imidazole]. The eluted protein was dialyzed against the dialysis buffer [20 mM Hepes (pH 7.5) and 150 mM NaCl] using a 10-kDa cutoff dialysis tubing (Thermo Fisher Scientific) at 4°C for 16 hours. Tsi3 and His6 tags were cleaved off by the HRV-3C protease during the dialysis and removed by applying the protein solution after overnight dialysis onto an INDIGO-Ni resin. The flow through containing IGFBP7 was collected and concentrated with a 10-kDa cutoff concentrator (Millipore), loaded onto a Superdex 200 increase 10/300 GL size exclusion column (Cytiva), and eluted with the SEC buffer (20 mM Hepes at pH 7.5 and 150 mM NaCl). The fractions containing IGFBP7 proteins were identified by SDS–polyacrylamide gel electrophoresis (PAGE) and pooled (fig. S1).

The purification procedure of WT and mutant IGFBP7-eGFP was similar to that of IGFBP7 except IGFBP7-eGFP was subject to SEC purification immediately after HRV-3C protease cleavage without removing the Tsi3 and His6 tags using INDIGO-Ni affinity chromatography. The SEC fractions containing IGFBP7-eGFP proteins were identified by SDS-PAGE and pooled.

To make IGFBP7/heparin complex for cryo-EM analyses, heparin sodium salt from porcine intestinal mucosa (Sigma-Aldrich) was added to IGFBP7 at a molar ratio of 4:1 (heparin:IGFBP7). After incubation at 4°C for 30 min, the protein/heparin mixture was loaded onto a Superdex 200 increase 10/300 GL size exclusion column and eluted with the SEC buffer. The peak fraction was concentrated to 8 mg ml^−1^ using a 10-kDa cutoff concentrator (Millipore) and subject to cryo-EM grid preparation. All purification and following steps were performed at 4°C or on ice.

The purification of HGF used in the c-MET activation assay was performed as described in a previous study (*69*). Briefly, the pEZT-HGF plasmid was transformed into DH10Bac bacteria (Geneva Biotech) to generate bacmid DNA. Recombinant baculovirus was produced by transfecting Sf9 cells with the bacmid DNA using Cellfectin (Invitrogen). HGF protein was expressed in HEK293F cells by infecting cells at a virus-to-cell ratio of 1:10 (v/v). Following infection, cultures were supplemented with 5 mM sodium butyrate and 5% fetal bovine serum (FBS; Gibco). Cells were cultured for 96 hours at 37°C in 8% CO_2_. Culture medium containing secreted HGF was harvested by centrifugation and clarified by filtration through a glass fiber filter. The clarified medium was adjusted to a final concentration of 50 mM sodium/potassium phosphate (pH 6.5) and applied to a cation-exchange column (HiTrap SP HP, Cytiva). Bound protein was eluted using a linear gradient up to 50% buffer B [20 mM sodium/potassium phosphate (pH 6.5) and 2 M NaCl]. The eluated protein was further purified by SEC using a Superose 6 Increase 100/30 column (Cytiva) with buffer containing 20 mM Hepes (pH 7.5) and 400 mM NaCl.

### Protein cross-linking

The chemical cross-linking reaction using the cross-linker BS3 (Thermo Fisher Scientific) was started by mixing WT or mutant IGFBP7 (1 mg/ml) with or without heparin in cross-linking buffer (20 mM Hepes at pH 7.5 and 150 mM NaCl or HN buffer) to a 50-fold molar excess of BS3. BS3 was freshly dissolved in HN buffer. The reaction was incubated at room temperature for 30 min and subsequently quenched by 1 M tris-HCl (pH 7.5). The cross-linked products were analyzed by SDS-PAGE.

### Cell aggregation assay

The cell aggregation assay was performed following published protocols with modifications (*79*). HEK293T cells were seeded in a 6-well plate (Corning) containing 2 ml of Dulbecco’s modified Eagle’s medium (DMEM; Thermo Fisher Scientific) supplemented with 10% (v/v) FBS (Thermo Fisher Scientific) and incubated overnight at 37°C (*44*). When cells reached 70 to 80% confluency, the media were aspirated and replaced with Opti-MEM reduced serum media, and the cells were transfected with 500 ng of pcDNA3.1+GFP using threefold excess (w/w) of Lipofectamine 2000 Reagent (Thermo Fisher Scientific). After 6 hours of incubation at 37°C, the media Opti-MEM reduced serum media were aspirated and replaced with DMEM supplemented with 10% FBS, and purified WT or mutant IGFBP7 was added to each well of the plate to a final concentration of 100 nM. After overnight incubation, the media were aspirated, the cells were washed with 500 μl of 1x Dulbecco’s phosphate-buffered saline (DPBS) and detached with 1x DPBS containing 1 mM EGTA and supplemented with 15 μl of DNase I (50 mg ml^−1^; Sigma-Aldrich). Cells were resuspended by pipetting and transferred to an Eppendorf tube, and additional 10 μl of DNase solution (50 mg ml^−1^) was added to each sample. Cells were counted using a Countess 3 cell counter (Thermo Fisher Scientific), and an appropriate number of cells were added to 400 μl of incubation solution (DMEM supplemented with 10% FBS, 10 mM CaCl_2_, and 10 mM MgCl_2_) in a noncoated 24-well plate. Additional 100 nM purified WT or mutant IGFBP7 was added to each sample, and cells were then placed on a shaker at 120 rpm at 30°C for 15 min and imaged using a ZOE Fluorescent Cell Imager (Bio-Rad) equipped with a 10× objective. Both the white-field (WF) and fluorescence (FL) images were recorded; however, because of better contrast, only fluorescence images were used for cell aggregation quantification. The resulted fluorescence images were analyzed using Fiji ImageJ (version 2.16.0), and cell aggregation areas were calculated using an in-house ImageJ macro script. The *P* values of the data were calculated using a two-way analysis of variance (ANOVA) in Prism 10 (GraphPad).

### Cell surface binding of IGFBP7

HEK293T cells were seeded in Lab-Tek 4-well Chambered Coverglass plate containing 600 μl of DMEM supplemented with 10% FBS and incubated overnight at 37°C. When cells reached ∼40% confluency, purified WT or mutant IGFBP7-eGFP was added to each well to a final concentration of 100 nM. After incubation overnight at 37°C, the media were aspirated and gently washed twice with 300 μl of DPBS to remove the unbound IGFBP7-eGFP. Subsequently, 600 μl of DMEM supplemented with 10% FBS was added back to each well, and cells were incubated at 37°C for 30 min and imaged under a Zeiss LSM 780 inverted confocal microscopy with a 20x objective. Images were processed and analyzed using Fiji ImageJ (version 2.16.0).

### Cell-based IR and c-MET activation assay

The IR and c-MET activation assays were performed as previously described (*77*, *80*–*83*). The cDNAs of full-length IR or c-MET were cloned into the pCS2 vector with a MYC or FLAG tag at the C terminus, respectively, and used for cell-based assays. HEK293T cells were seeded in 6-well plates containing 2 ml of DMEM supplemented with 10% FBS and incubated overnight at 37°C. When cells reached 70 to 80% confluency, 500 ng of plasmids was transfection to the cells using PEI with a PEI:DNA ratio of 3:1 (w/w). After 1 day, the cells were serum starved using FBS-free DMEM for 16 to 18 hours. Serum-starved cells were treated with various amount IGFBP7 for 2 hours, followed by stimulation of indicated concentrations of insulin (INS; for IR activation) or HGF (for c-MET activation). Depending on the experimental conditions, the cells were treated with various amount IGFBP7, insulin, or HGF, and the duration of stimulation also varies in time-dependent experiments. After treatment, cells were incubated with cell lysis buffer containing 50 mM Hepes (pH 7.4), 150 mM NaCl, 10% (v/v) glycerol, 1% (v/v) Triton X-100, 1 mM EDTA, 5 mM dithiothreitol (DTT), and 2 mM phenylmethylsulfonyl fluoride (PMSF) for 1 hour. After centrifugation at 16,000*g* at 4°C for 10 min, cell lysates were analyzed by SDS-PAGE and Western blotting. For IR activation assays, anti-IR-pY1150/1151 (Cell Signaling) and anti-Myc (Cell Signaling) were used as primary antibodies, and anti-rabbit immunoglobulin G (IgG) (Cell Signaling) was used as secondary antibody. For c-MET activation assays, anti-MET-pY1234/1235 (Cell Signaling) and anti-FLAG (Sigma-Aldrich) were used as primary antibodies, and anti-rabbit IgG and anti-mouse IgG were used as secondary antibodies. The membranes were treated with the SuperSignal West Dura Extended Duration Substrate (Thermo Fisher Scientific) and imaged with the ChemiDoc MP Imaging System (Bio-Rad).

### SEC coupled with multiangle light scattering

The SEC-MALS experiment was conducted at 4°C in SEC-MALS buffer [20 mM Hepes (pH 7.6) and 200 mM NaCl]. This buffer was used to exhaustively equilibrate the chromatographic column (a Superdex 200 Increase 10/300 GL column, connected to an Akta Pure system, Cytiva) before the experiments commenced. Just before injection (loop volume: 100 μl) onto the column at a flow rate of 0.5 ml/min, the samples were filtered through a 0.22 μm spin filter. The eluate was detected with a Cytiva UV detector and TREOS light-scattering detector (Wyatt Technologies). Data for bovine serum albumin (2 mg/ml; Pierce) were also acquired, and the monomer peak was used for instrument calibration. Data were analyzed with Wyatt’s ASTRA software version 7.1.0.29. Extinction coefficients for the analyses were calculated using the amino acid sequence according to the formula $\frac{\#\;\text{Tyr}\;\times \;\left(1490\right)\;+\;\#\;\text{Trp}\;\times \;\left(5500\right)\;+\;\#\;\text{Cystine}\;\times \;\left(125\right)}{M}$, where *M* is the molecular weight of the protein in Da.

### BLI assay

The binding affinities between heparins and IGFBP7 were determined by BLI. Before the BLI experiments, three heparin molecules—LCHS (Sigma-Aldrich), 12HO (Iduron Ltd.), and tinzaparin (Sigma-Aldrich)—were biotinylated using the EZ-Link Hydrazide-LC-Biotin (Thermo Fisher Scientific) according to the manufacturer’s instructions. Briefly, heparins (2 mg/ml) were oxidized with 5 mM sodium *meta*-periodate (Thermo Fisher Scientific) for 30 min at 4°C in the dark in oxidation buffer [0.1 M sodium acetate (pH 5.5)]. Excess sodium *meta*-periodate was removed by gel filtration using Zeba Spin Desalting Columns (Thermo Fisher Scientific) preequilibrated with PBS buffer. The oxidized heparins were then reacted with 5 mM EZ-Link Hydrazide-LC-Biotin for 2 hours at room temperature. Excess biotin was subsequently removed by gel filtration through Zeba Spin Desalting Columns.

BLI experiments were performed using an Octet R2 system (Sartorius). Biotinylated heparins diluted in BLI buffer [20 mM Hepes (pH 7.6), 150 mM NaCl, and 0.02% Tween 20)] were immobilized onto SA biosensors (Sartorius, 18-0009) for 240 s, followed by a 180-s baseline equilibration in BLI buffer. WT or mutant IGFBP7 proteins were then associated with the heparin-coated biosensor surfaces for 180 s, followed by a 240-s dissociation phase. Association and dissociation curves were recorded at 30°C using the standard kinetics acquisition rate (5.0 Hz with averaging by 20). Data were processed using the Octet Analysis Studio software (Sartorius), and dissociation constants (*K*_d_) were obtained by global fitting analysis.

### Cryo-EM grid preparation

Immediately before preparing the cryo-EM grids, 2 mM fluorinated Fos-Choline-8 (Anatrace) was added to the IGFBP7/heparin sample to prevent the protein from being damaged by the hydrophobic air-water interface on a cryo-EM grid. Four microliters of the IGFBP7/heparin complex at 8 mg/ml was applied to glow-discharged Quantifoil R1.2/1.3 300-mesh gold holey carbon grids (Quantifoil, Micro Tools GmbH, Germany). Grids were blotted under 100% humidity at 4°C and plunge frozen in liquid ethane using a Mark IV Vitrobot (Thermo Fisher Scientific).

### Cryo-EM data collection and image processing

EM data acquisition, image processing, model building, and refinement were performed following previous protocols with some modifications (*84*). Micrographs were collected in the counting mode on Titan Krios microscopes (Thermo Fisher Scientific) with K3 Summit direct electron detectors (Gatan). The nominal magnification and pixel size of each dataset are summarized in Table 1. Motion correction and dose weighting of the micrographs were carried out using the Motioncor2 program (version 1.2) (*85*). GCTF 1.06 was used for CTF correction (*86*). Template-based particle picking was carried out using the autopick tool in RELION 5.0 (*87*). Particles were binned four times and cleaned up with multiple rounds of 2D and 3D classification in RELION. The exact procedures are summarized in supplementary figures. The initial mode for 3D classification and refinement was generated using the SGD method in RELION. The final focused refinement on the central 12 IgC2 domains was carried out with D6 symmetry, and the refined maps were further improved by using Bayesian polishing, blush regularization, and CTF refinement at the final stage. Subsequently, we performed the 3D classification with small angle sampling search and D6 symmetry expansion to improve the density for the N-terminal IB/Kazal domains (3.7° sampling, 20° searching range; C1 symmetry). The resulting cryo-EM map of the entire IGFBP7 barrel was resolved at a 4.5-Å resolution. The Fourier shell correlation (FSC) 0.143 criterion was used for estimating the resolution of the maps. Local resolution was calculated in RELION.

**Table 1.** Cryo-EM data collection and structure refinement statistics. RMS, root mean square.

Structure	Dodecamer of the IgC2 domain	Dodecamer of intact IGFBP7
Magnification	105,000	105,000
Voltage (kV)	300	300
Electron exposure (e^−^/Å^2^)	60	60
Defocus range (μm)	1.5–2.5	1.5–2.5
Pixel size (Å)	0.826	0.826
Symmetry imposed	D6	C1
Initial particle images (no.)	2,961,957	2,961,957
Final particle images (no.)	26,016	31,657
Map resolution (Å)	3.4	4.5
FSC threshold	0.143	0.143
		
Initial model (AlphaFold model)	AF-Q16270-2-F1	AF-Q16270-2-F1
Model resolution (Å)	3.4	4.5
FSC threshold	0.143	0.143
Map sharpening *B* factor (Å^2^)	−40	−30
Model composition		
Nonhydrogen atoms	10,032	20,904
Protein residues	1320	2904
Protein *B* factors (Å^2^)	84.6	379
RMS deviations		
Bond length (Å)	0.002	0.003
Bond angle (°)	0.526	0.595
Validation		
MolProbity score	0.89	1.28
Clashscore	1.5	3.3
Poor rotamers (%)	0.9	1
Ramachandran plot		
Favored (%)	99	97.2
Allowed (%)	1	2.8
Outliers (%)	0	0

### Model building and refinement

Model building was initiated by docking of the AlphaFold predicted models of the individual proteins into the cryo-EM maps. The models were adjusted manually in Coot and refined using the ISOLDE plugin in ChimeraX (*88*, *89*). The coordinates and atomic *B* factors of the models were further refined using the real-space refinement module in Phenix (*90*), with secondary structure and Ramachandran restraints. The validation of the models was carried out using the MolProbity module in Phenix.
