# Supplementary material for: Insulin-like growth factor–binding protein 7 is a soluble cell adhesion molecule
Source: Sci Adv. 2026 Aug 26;12(35):eaeg1148. doi: 10.1126/sciadv.aeg1148 (PMC13510594; doi:10.1126/sciadv.aeg1148)
Supplement: Supplementary file 1 [file sciadv.aeg1148_sm.pdf]

Science Advances
AAAS

Supplementary Materials for

Insulin-like growth factor–binding protein 7 is a soluble cell adhesion molecule

Kai Cai *et al.*

Corresponding author: Xuewu Zhang, xuewu.zhang@utsouthwestern.edu;
Xiao-chen Bai, xiaochen.bai@utsouthwestern.edu

Sci. Adv. **12**, eaeg1148 (2026)
DOI: 10.1126/sciadv.aeg1148

This PDF file includes:

Figs. S1 to S9

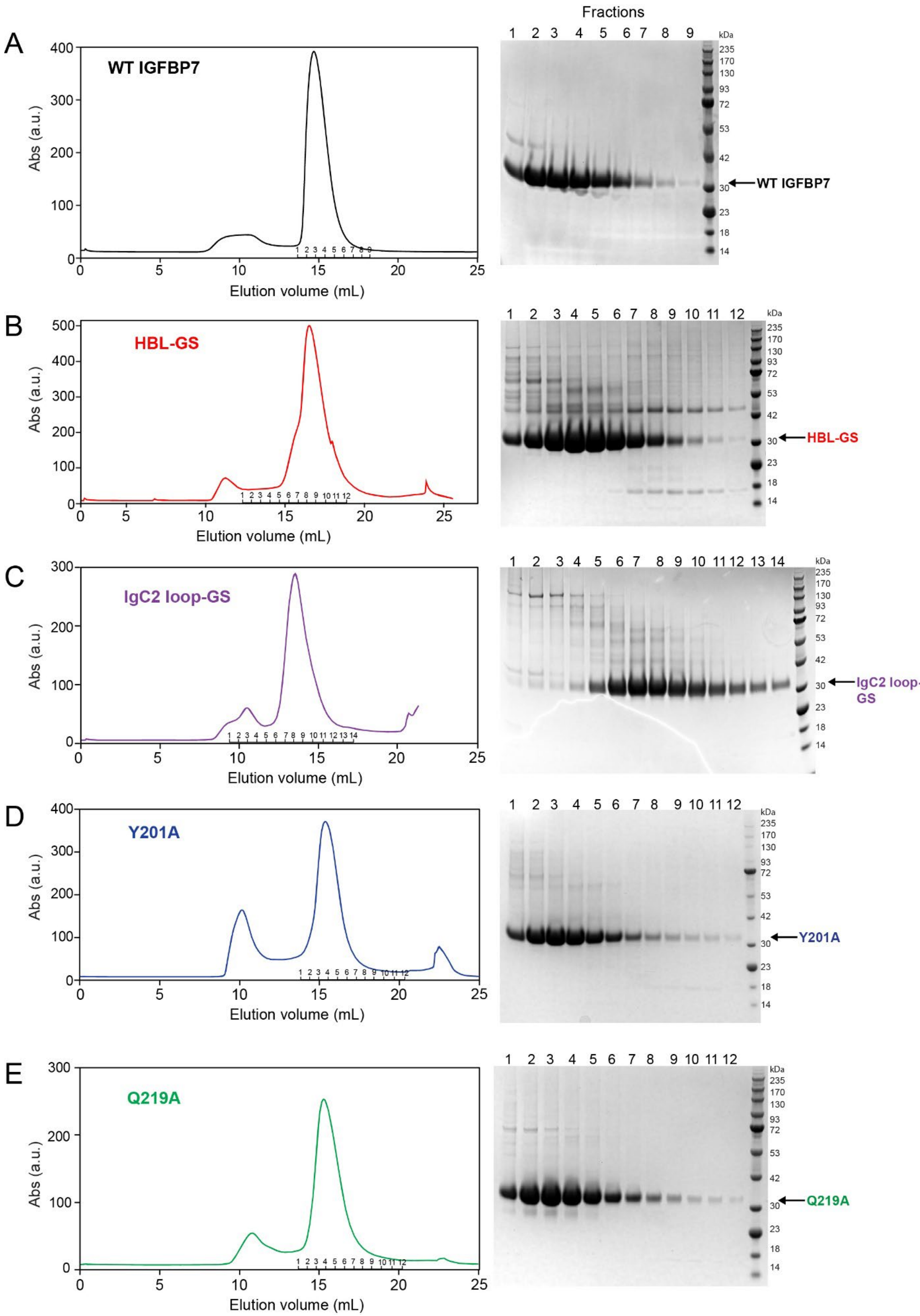

Fractions
A
WT IGFBP7
Abs (a.u.)
Elution volume (mL)
kDa
235
170
130
93
72
53
42
30
23
18
14
WT IGFBP7
B
HBL-GS
HBL-GS
C
IgC2 loop-GS
IgC2 loop-GS
D
Y201A
Y201A
E
Q219A
Q219A

Fig. S1.

Purification of IGFBP7. Representative size-exclusion chromatography (SEC) profiles and the corresponding sodium dodecyl sulfate polyacrylamide gel electrophoresis (SDS-PAGE) analyses results of IGFBP7. (**A**) wild type (WT) IGFBP7, (**B**) heparin binding loop mutant (HBL-GS), (**C**) IgC2 loop mutant (IgC2 loop-GS), (**D**) Y201A, and (**E**) Q219A.

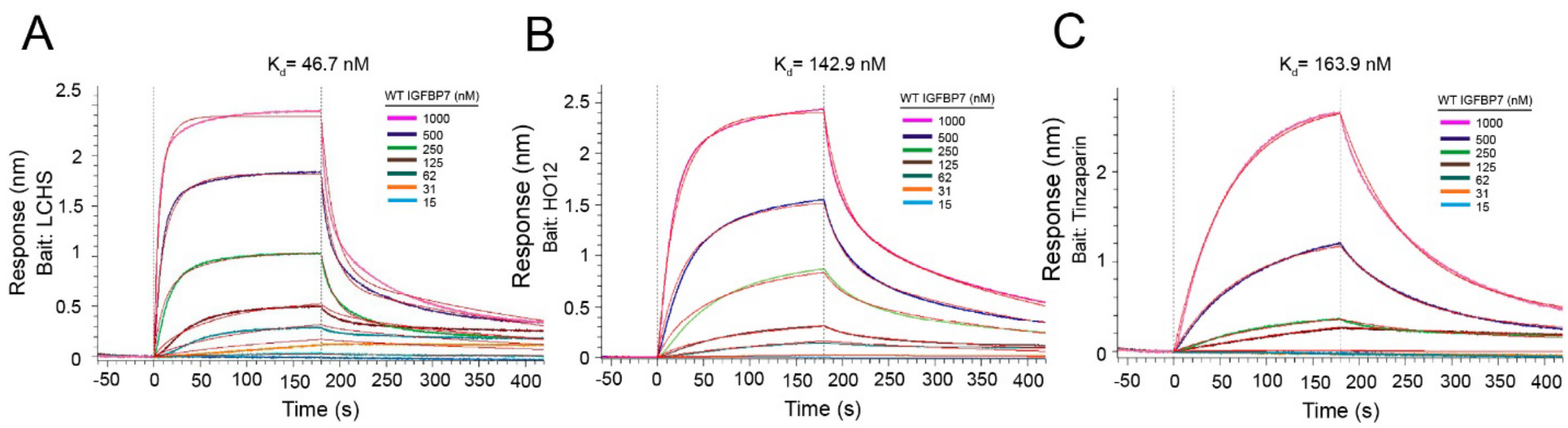

Fig. S2.

BLI measurement of binding between WT IGFBP7 and three heparins with varied chain lengths: (**A**) LCHS, (**B**) HO12 and (**C**) tinzaparin. Biotinylated heparins were immobilized onto the Streptavidin (SA) sensors as bait molecules. The concentration of WT IGFBP7 and calculated dissociate constant (K_d) are indicated in figures.

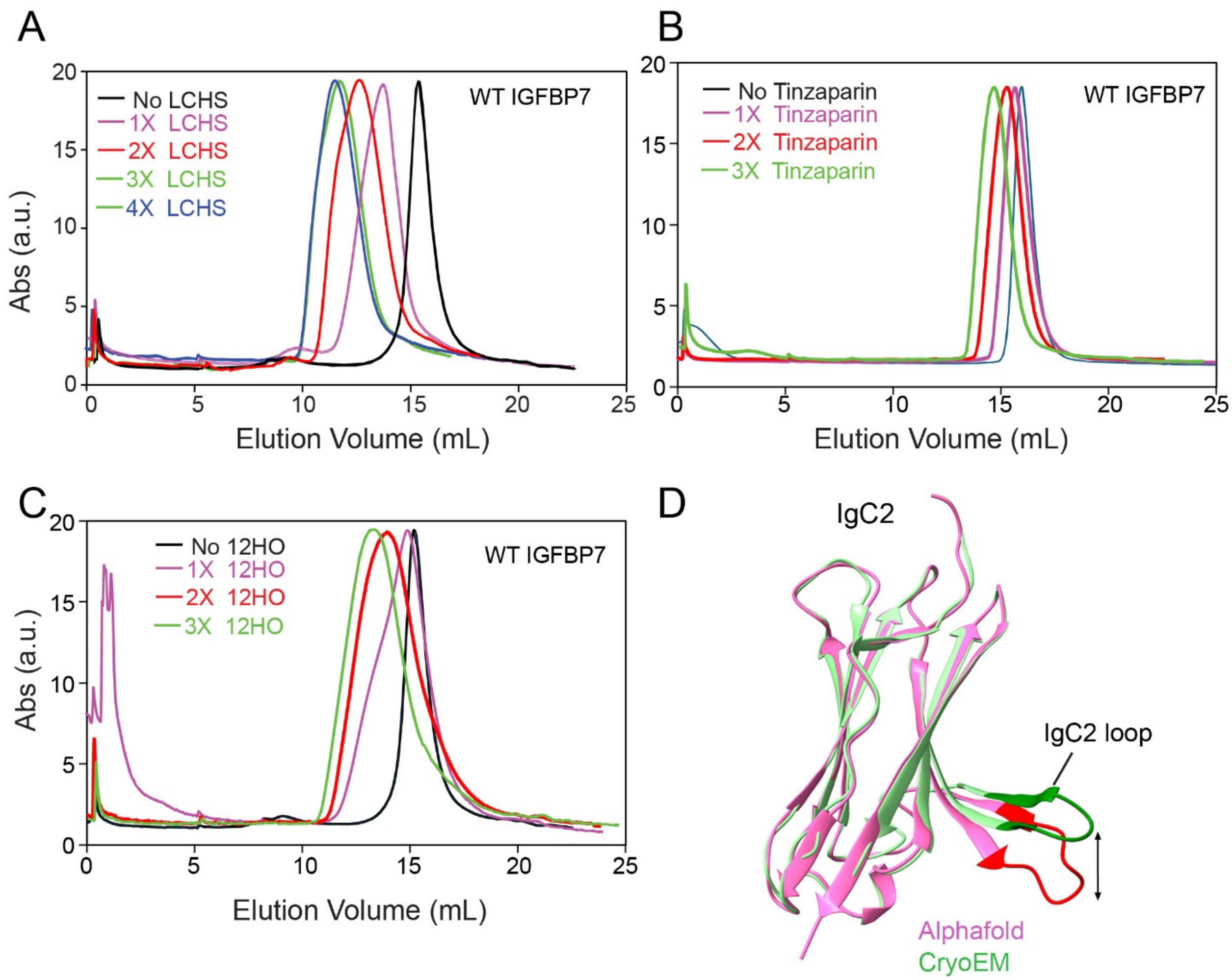

Fig. S3.
(**A-C**) SEC profiles of WT IGFBP7 in the presence of various amounts of heparin with different chain lengths. LCHS, long-chain heparin; 12HO, heparin dodecasaccharide; tinzaparin. (**D**) Comparison of the IgC2 domain structures predicted by Alphafold (pink) and in the cryo-EM structure of IGFBP7 homo-dodecamer (green). The IgC2 loop adopts a different conformation in the two structural models.

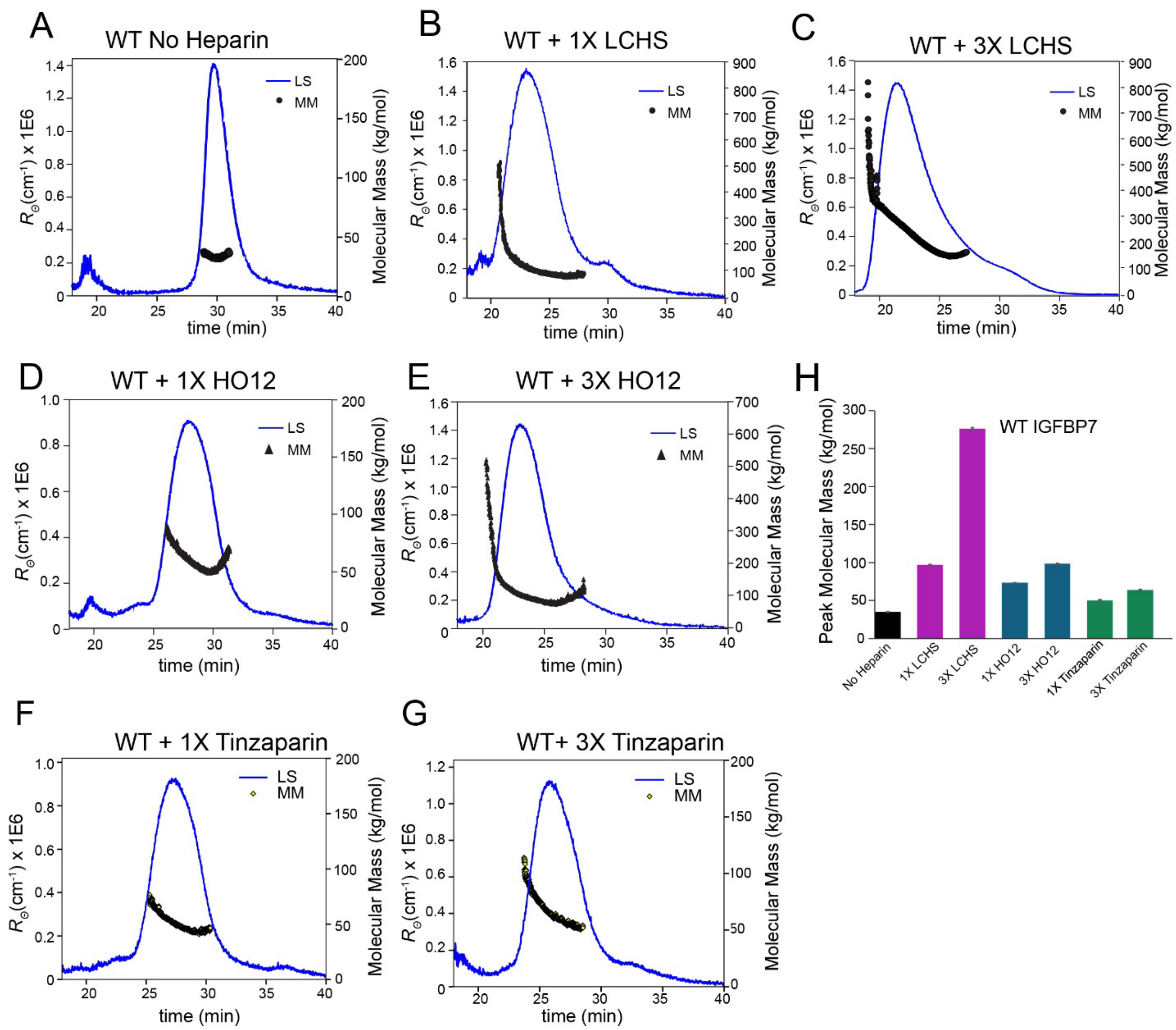

Fig. S4.
SEC-MALS profiles of WT IGFBP7 in the presence of different amounts of heparins with varying chain lengths (LCHS, 12HO, and tinzaparin). Solid lines represent light scattering signals in physical units (left axis), and markers indicate the corresponding molar masses (right axis). LS, light scattering; MM, molar masses. **(A-C)** WT IGFBP7 (**A**) alone, (**B**) with an equimolar amount of LCHS, or (**C**) with a 3-fold molar excess of LCHS. **(D-E)** WT IGFBP7 (**D**) with an equimolar amount of 12HO, or (**E**) with a 3-fold molar excess of 12HO. (**F-G)** WT IGFBP7 (**F**) with an equimolar amount of tinzaparin, or (**G**) with a 3-fold molar excess of tinzaparin. (**H**) Comparison of the peak molecular masses from SEC-MALS profiles of WT IGFBP7 under the specified conditions.

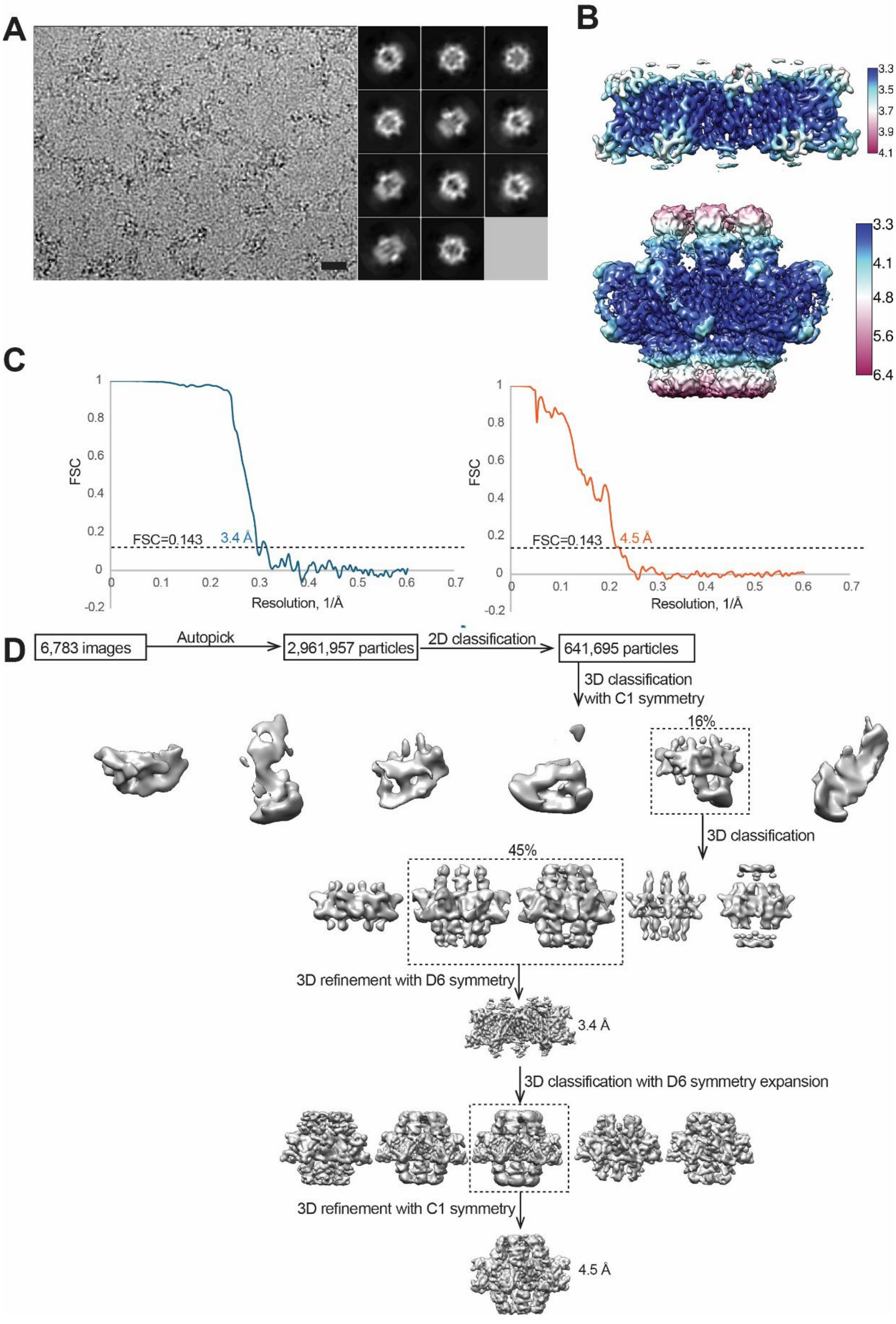

Fig. S5.

The workflow of cryo-EM data processing. Scale bar: 20 nM.

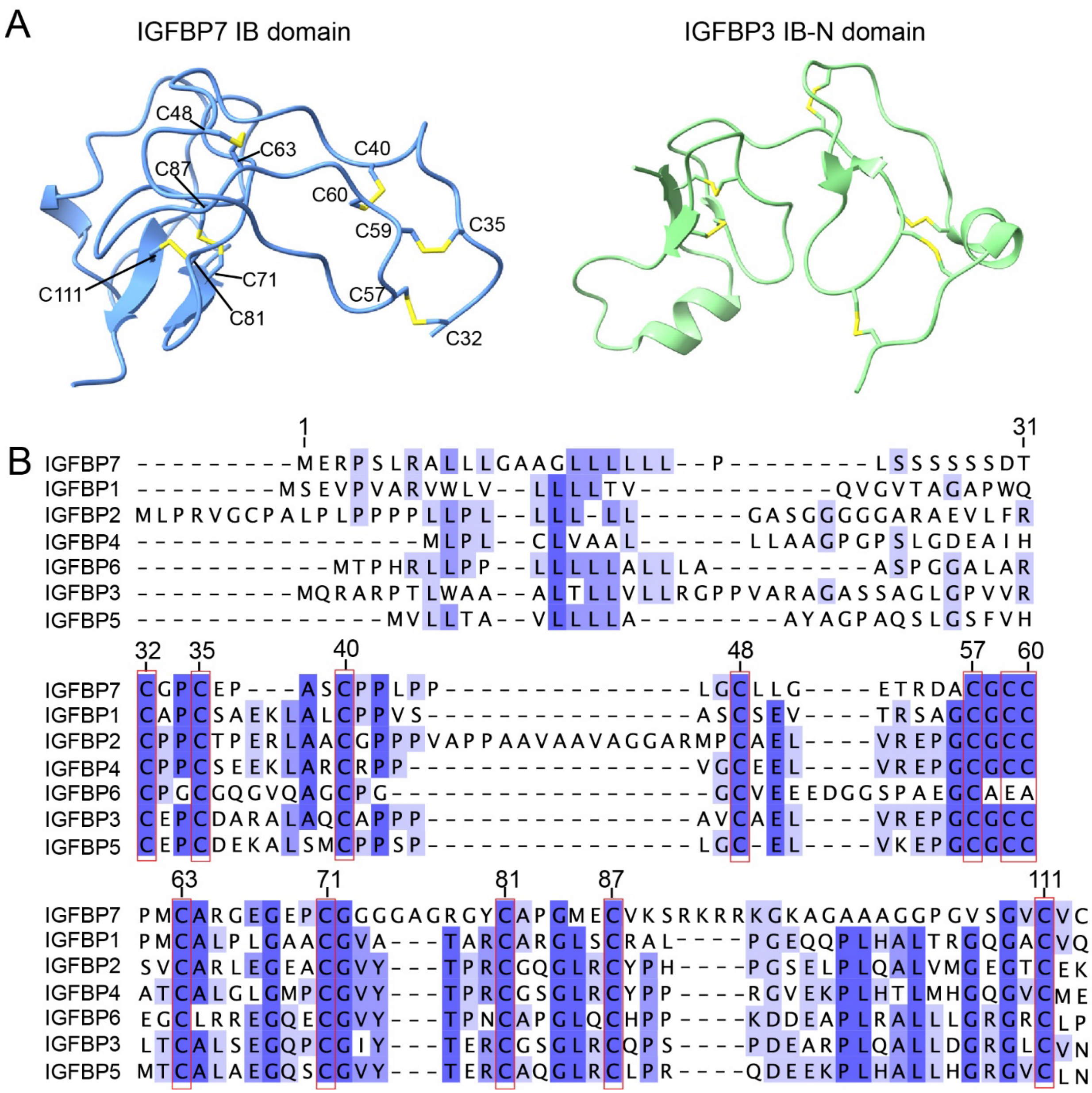

Fig. S6.

(**A**) Structures of IB domain of IGFBP7 and IB-N domain of IGFBP3 (PDB:7WRQ). The six disulfide bonds in both structures are highlighted with stick representations. (**B**) Multiple sequence alignment of IGFBP family proteins. Amino acid positions are numbered according to the IGFBP7 sequence. The conserved cysteine residues are highlighted with red boxes. Color code: blue, conserved; light blue, partially conserved.

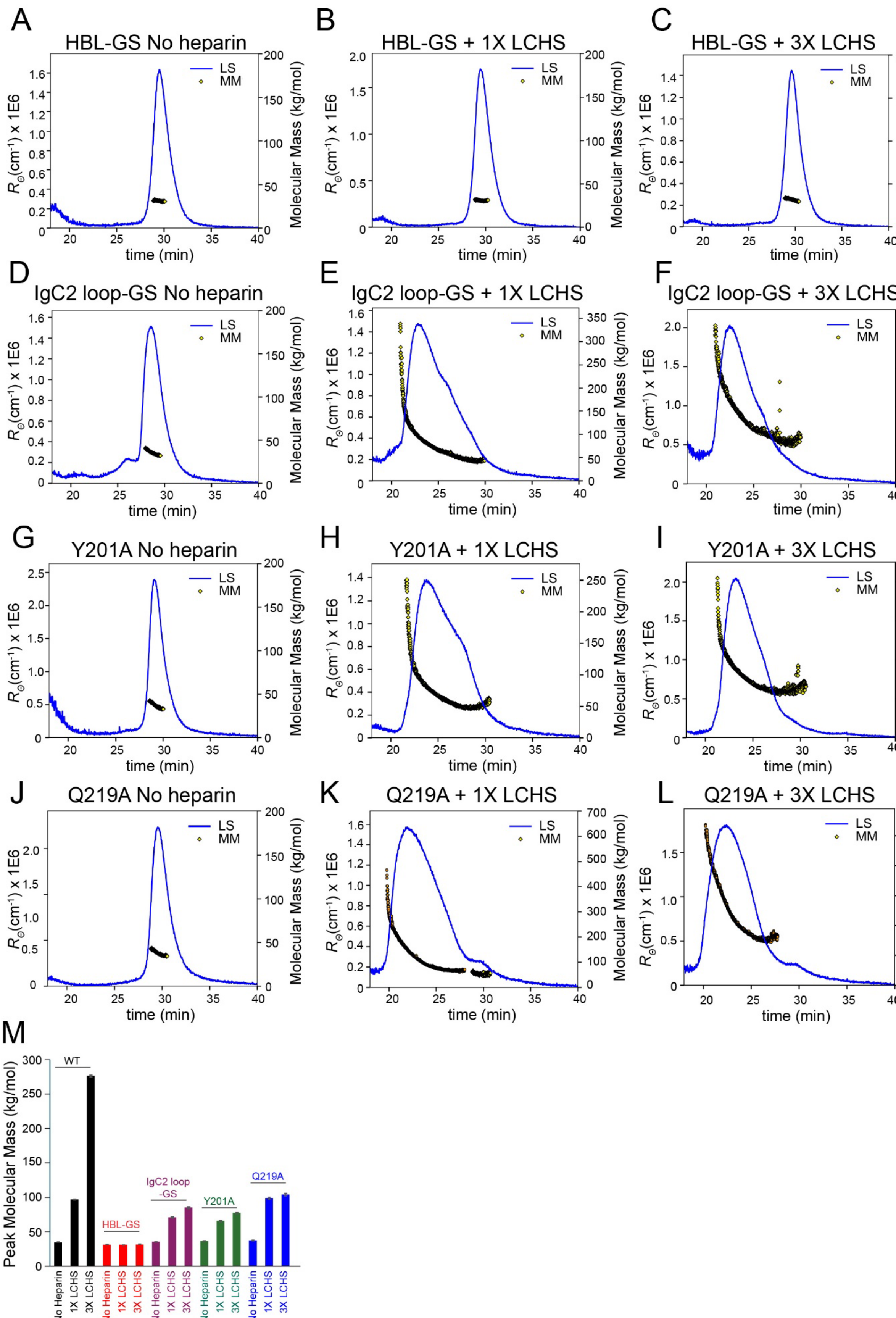

A
HBL-GS No heparin
B
HBL-GS + 1X LCHS
C
HBL-GS + 3X LCHS
D
IgC2 loop-GS No heparin
E
IgC2 loop-GS + 1X LCHS
F
IgC2 loop-GS + 3X LCHS
G
Y201A No heparin
H
Y201A + 1X LCHS
I
Y201A + 3X LCHS
J
Q219A No heparin
K
Q219A + 1X LCHS
L
Q219A + 3X LCHS
LS
MM
R_Θ(cm^{-1}) x 1E6
Molecular Mass (kg/mol)
time (min)
M
Peak Molecular Mass (kg/mol)
WT
HBL-GS
IgC2 loop -GS
Y201A
Q219A
No Heparin
1X LCHS
3X LCHS

Fig. S7.

SEC-MALS profiles of mutant IGFBP7 in the presence of various amount heparin LCHS. Solid lines represent the light scattering in physical units (left axis), and markers show the respective molar masses (right axis). LS, light scattering; MM, molar masses. (**A**-**C**) HBL-GS IGFBP7 mutant: (**D**) alone, (**E**) with an equimolar amount of LCHS, or (**F**) with a 3-fold molar excess of LCHS. (**D**-**F**) IgC2-GS IGFBP7 mutant: (**G**) alone, (**H**) with an equimolar amount of LCHS, or (**I**) with a 3-fold molar excess of LCHS. (**G**-**I**) Y201A IGFBP7 mutant: (**J**) alone, (**K**) with an equimolar amount of LCHS, or (**L**) with a 3-fold molar excess of LCHS. (**J**-**L**) Q219A IGFBP7 mutant: (**M**) alone, (**N**) with an equimolar amount of LCHS, or (**O**) with a 3-fold molar excess of LCHS. (**M**) Comparison of the peak molecular masses from SEC-MALS profiles of WT or mutant IGFBP7 under the specified conditions.

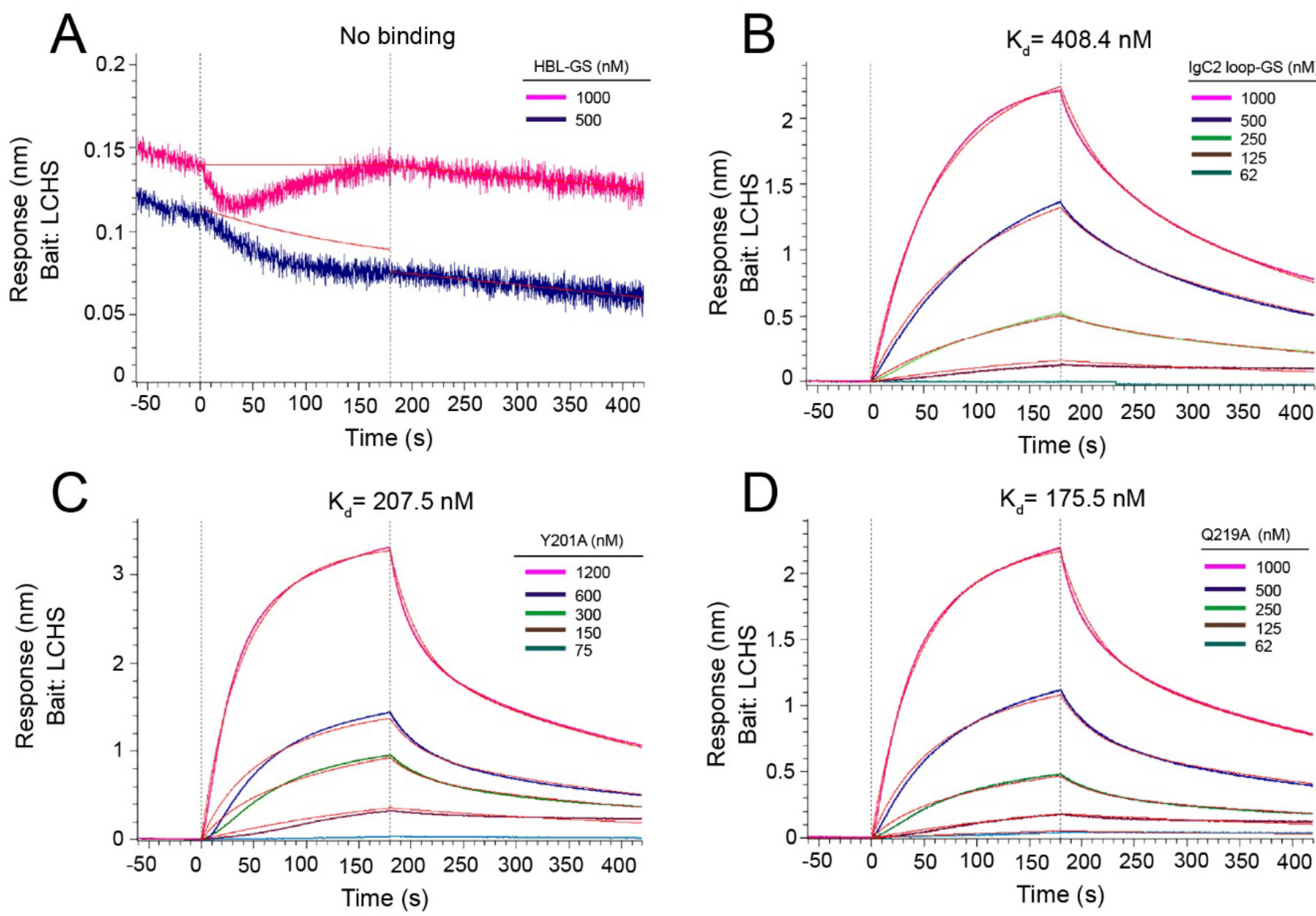

Fig. S8.
BLI measurement of binding between LCHS and IGFBP7 mutants: (**A**) HBL-GS, (**B**) IgC2-GS, (**C**) Y201A and (**D**) Q219A. Biotinylated heparins were immobilized onto the Streptavidin (SA) sensors as baits. The concentration of WT IGFBP7 and calculated dissociate constant (K_d) are indicated in figures.

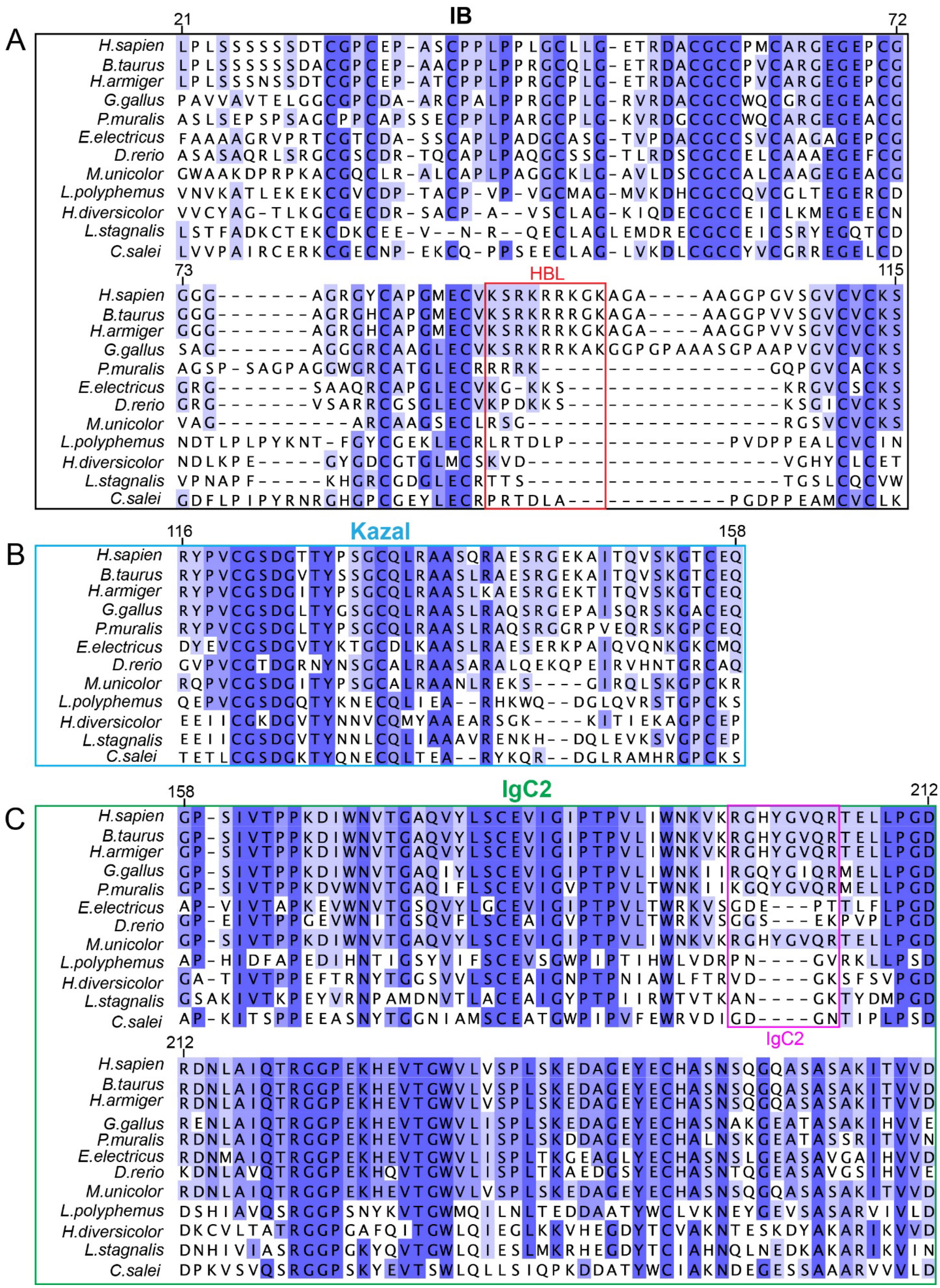

A
IB
21
72
73
115
HBL
B
Kazal
116
158
C
IgC2
158
212
212
IgC2
H.sapien
B.taurus
H.armiger
G.gallus
P.muralis
E.electricus
D.rerio
M.unicolor
L.polyphemus
H.diversicolor
L.stagnalis
C.salei

Fig. S9.

Multiple sequence alignment of IGFBP7 proteins across species. Amino acid positions are numbered according to the IGFBP7 sequence. (**A**) IB domain, (**B**) Kazal domain, (**C**) IgC2 domain. The heparin binding loop (HBL) and IgC2 loop are highlighted with red and magenta boxes, respectively. Color code: blue, conserved; light blue, partially conserved. *H.sapien, Homo sapiens (human); B.taurus, Bos taurus (cattle); H.armiger, Hipposideros armiger (great roundleaf bat); G.gallus, Gallus gallus (chicken); P.muralis, Podarcis muralis (common wall lizard); E.electricus, Electrophorus electricus (electric eel); D.rerio, Danio rerio (zebrafish); M.unicolor, Microcaecilia unicolor (unicolored caecilian); L.polyphemus, Limulus polyphemus (horseshoe crab); H.diversicolor, Helice diversicolor (mudflat crab); L.stagnalis, Lymnaea stagnalis (great pond snail); C.salei, Cupiennius salei (huntsman spider).*
